# Editorial: Nutrition and sustainable development goal 12: responsible consumption

**DOI:** 10.3389/fnut.2024.1394417

**Published:** 2024-03-22

**Authors:** Muhammad Bilal Sadiq, Luca Secondi, Elena Velickova, João Miguel Rocha, Laura Rossi, Ximena Schmidt Rivera, Graziana Difonzo, Monica Rosa Loizzo, Fatih Ozögul

**Affiliations:** ^1^School of Life Sciences, Forman Christian College (A Chartered University), Lahore, Pakistan; ^2^Department for Innovation in Biological, Agri-food and Forestry Systems, University of Tuscia, Viterbo, Italy; ^3^Faculty of Technology and Metallurgy, Saints Cyril and Methodius University of Skopje, Skopje, North Macedonia; ^4^Centro de Biotecnologia e Química Fina – Laboratório Associado (CBQF), Escola Superior de Biotecnologia, Universidade Católica Portuguesa, Porto, Portugal; ^5^Laboratory for Process Engineering, Environment, Biotechnology and Energy (LEPABE), Faculty of Engineering, University of Porto, Porto, Portugal; ^6^Associate Laboratory in Chemical Engineering (ALiCE), Faculty of Engineering, University of Porto, Porto, Portugal; ^7^Council for Agricultural Research and Economics-Research Centre for Food and Nutrition (CREA Food and Nutrition), Rome, Italy; ^8^Department of Chemical Engineering, College of Engineering, Design and Physical Sciences, Brunel University London, Uxbridge, United Kingdom; ^9^Department of Soil, Plant and Food Science, University of Bari Aldo Moro, Bari, Italy; ^10^Department of Pharmacy, Health and Nutritional Sciences, University of Calabria, Rende, Italy; ^11^Department of Seafood Processing Technology, Faculty of Fisheries, Cukurova University, Adana, Turkey; ^12^Biotechnology Research and Application Center, Cukurova University, Adana, Turkey

**Keywords:** food waste, sustainable consumption, sustainable development goals, food supply chain, consumer awareness

Food is the core element of sustainability and it is an integral component of sustainable development goals (SDGs). The food system contributes between 25–34% of the global greenhouse gas emission, together with other detrimental environmental impacts including biodiversity loss, acidification of soils, etc. Approximately, 30% of the produced food is lost or wasted on a global scale, which involves postharvest loss, processing and distribution loss and wastage at a consumer level. A rapid rise in global population and food losses collectively compromise food security and SDGs. The effective management of food waste and byproducts can minimize the challenges of food security and environmental hazards associated with the disposal of food waste. Implementation of effective strategies and control measures can reduce food loss and enhance food availability. These strategies include integrated supply chain models, raising awareness, redistribution, effective models for recovery of value-added products, and management of disposals. Among SDGs, the target of SDG 12 (sustainable consumption and production) is addressed to significantly reduce food waste at all levels of food supply chain i.e., farm to fork.

## Food loss and waste

On a global scale food loss and waste (FLW) is a major hurdle in achieving sustainable food systems. A food system involves all stages of the supply chain i.e., from farm to fork. Around one-third proportion of globally produced food is lost or wasted. Food loss corresponds to post-harvest loss, whereas food waste is primarily associated with the consumer level (1). The term food loss and waste highlight all the food resources including byproducts, secondary metabolites and all other edible components which can be used as a food or can enter food chain but either lost or wasted during preharvest, post-harvest, processing, distribution, and consumption levels. The magnitude of food waste generated is usually self-reported and based on predictions. Comprehensive analyses about economic, environmental and recycling impacts of food waste are extremely important for developing FLW management policies as highlighted in the systematic review by Bilali et al.

## Contributing factors of food loss and waste and control strategies

The globalization and industrialization of food processing, high demand for animal protein, changes in eating habits and lifestyle are major hurdles toward the achievement of sustainable food system. In developing countries, a major proportion of food is wasted at postharvest level due to lack of infrastructure and technology, whereas in developed countries consumer level wastage predominates (2). Tonini et al. reported that lack of food management and excessive purchase of perishable foods are predominant reasons of food waste in households with children. It is important to identify the potential factors and magnitude of food waste generated at each scale, i.e., at production, processing distribution and consumer levels. The implementation of audit and accountability can contribute toward minimization of food waste. Cook et al. in their study identified the important factors which influence the implementation of food waste audits in foodservices.

Due to diversified nature of food, the contributing factors for FLW can be lack of an appropriate technology, infrastructure, seasonal variability, variations in supply and demand, poor logistics, poor packaging, and storage (3). In their study Afriyie et al. highlighted the importance of food storage at consumer level. Consumer awareness and basic training about food storage can ensure food security and safety. FLW can be minimized by effective food supply chain management (SCM), raising awareness, improved logistic framework, digitalization of supply chain and sharing responsibility with different stakeholders of the entire food chain.

## Sustainable consumption and recovery of value-added components

A strong coordination among all stakeholders is the backbone of sustainable food consumption model. The development of sustainable food SCM requires integration between the concepts of sustainability and SCM (4). The modification of dietary habits toward the use of organic fruits and vegetables, fiber enriched foods and exploration of alternative protein sources can contribute to sustainable food systems. For food policy makers, tools based on information, market and regulatory aspects are integral to developing a sustainable system (5). As mentioned by Grant and Rossi (a), there is dire need to develop national platforms to support the awareness programs and implementation of FLW management policies.

Lange and Nakamura proposed that edible insects can play a role in sustainable food systems due to the presence of high-quality protein, nutrients and associated environmental benefits. Food waste and byproducts can also be explored for valuable components, which can be utilized as functional ingredients in the food chain. Yeast protein is of great interest as an alternative protein due to ease of scale-up, relatively low-cost and nutritional value. Gärtner et al. in their study formulated vegan spread powders by using torula yeast as an alternative source of protein and observed variations in sensory acceptance with change geographical origin of the consumers. The food product development and marketing strategies should also consider consumer preferences based on cultural variations to avoid new food product failure.

The concept of food waste valorization was taken up by Chaklader et al. who reported that fish waste can be used as a source of functional protein hydrolysates, which can be used as a functional ingredient in aquafeed. The concept of responsible food consumption is directly linked with food waste prevention. In their study, Grant and Rossi (b) reported that consumers who follow the dietary guidelines are well aware of the concept of responsible consumption and limitations of food waste. Lack of adherence to dietary guidelines can comprise the health status of individuals. A similar concern was raised by Temesgen et al. who reported that approximately 80% of pregnant females in Ethiopia did not receive iron and folic acid supplements during required intervals due to non-adherence.

In summary, articles in this Research Topic highlight the importance of FLW and its impact on responsible consumption. To meet future needs and attain a sustainable food system, it is extremely important to implement the policies at national and global level to manage food waste, recover valuables from byproducts and ensure awareness among food chain stakeholders.

## Author contributions

MS: Conceptualization, Writing – original draft, Writing – review & editing. LS: Writing – review & editing. EV: Writing – review & editing. JR: Writing – review & editing. LR: Writing – review & editing. XS: Writing – review & editing. GD: Writing – review & editing. ML: Writing – review & editing. FO: Writing – review & editing.
